# Destructive invasion of the clavicle by desmoid tumor: a case report

**DOI:** 10.11604/pamj.2014.19.383.5693

**Published:** 2014-12-16

**Authors:** Guray Togral, Mustafa Turgut Yildizgoren, Murat Arikan, Safak Gungor

**Affiliations:** 1 Oncology Training and Research Hospital, Department of Orthopaedics and Traumatology, Ankara, Turkey; 2 Mustafa Kemal University, Faculty of Medicine, Department of Physical Medicine and Rehabilitation, Hatay, Turkey

**Keywords:** Desmoid tumor, clavicle, shoulder

## Abstract

Desmoid tumors are rare, soft-tissue neoplasms that do not metastasize, but exhibit aggressive growth and local invasion. They originate most frequently from abdominal fascial structures, although they can also appear at extra-abdominal sites. The most common extra-abdominal locations include the shoulder, chest wall, back, thigh, and head and neck. In children, desmoid tumors are more infiltrative, having a tendency towards osseous involvement more frequently than in adult patients. We report acase of a supraspinatus muscle desmoid tumor in a female patient with clavicle destruction.

## Introduction

Desmoid tumors (DTs) are benign, locally aggressive neoplasms that arise from connective tissue in muscle, fascia or aponeuroses [1]. The incidence of DTs has been reported as 2-4 cases per 1 million people [2]. DTs do not possess any metastatic potential but are locally aggressive. DTs typically invade skeletal muscle and adipose tissue in an infiltrative pattern; the presence of osseous invasion is not typical [3]. Although it is uncommon, DTs adjacent to osseous structures can cause a pressure effect and erosion of the cortex as well as a periosteal reaction [4]. In the present case, the patient is a 37-year-old woman with a DT that involves the supraspinatus muscle and is invading the clavicle, despite the relatively small size of the tumor.

## Patient and observation

A 37-year-old woman presented with a pain in her left shoulder of six month's duration. She had no history of trauma or neoplasm. On physical examination, her shoulder range of motion was full, with intact motor and sensory functions in the upper limb; however, the lateral edge of the clavicle was tender with palpation. Radiographs of the left shoulder showed an osteolytic lesion located at distal 1/3 of the left clavicle (Figure A). On MR images, an axial T1-weighted image, with fat suppression after intravenous gadolinium administration, revealed a mass measuring 2.0cm x 2.5cm within the supraspinatus muscle, with apparent invasion of the medulla of the clavicle (Figure B). During surgery to remove the tumor, severe destruction of the clavicle was noted (Figure C). The mass was removed via a wide excision (Figure D). Histopathology revealed a dense, collagenous neoplasm, with irregular margins and a diffuse infiltrative growing pattern. The cells consisted of uniform myofibroblasts with vesiculated nuclei and abundant amphophilic cytoplasm, with an absence of mitotic activity or necrosis. An immunohistochemical test for beta-catenin was positive, and microscopic analysis was revealed a diagnosis compatible with the characteristics of a desmoid tumor. There were no surgical complications and no recurrence was detected after 12 months. The patient is receiving follow-up care in our department.

**Figure 1:**
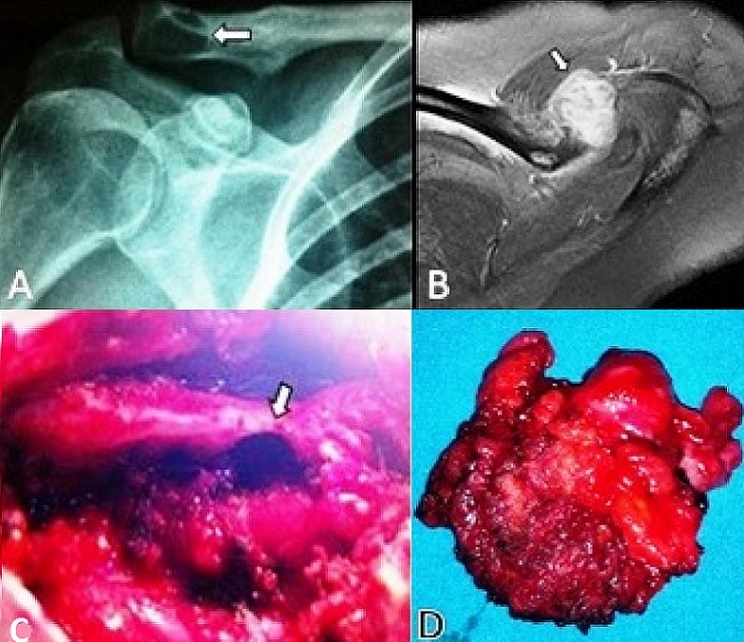
(A) plain radiograph of left shoulder demonstrates a radiolucent area at the lateral edge of the clavicle(arrow); (B) axial T1-weighted MR image with fat suppression after intravenous gadolinium administration shows an enhancing mass with cortical disruption and invasion into the medullary space (arrow); (C) the intra-op image shows severe osseous destruction of the clavicle (arrow); (D) macroscopic view of the excised desmoid tumor

## Discussion

Desmoid tumors (DTs)are rare, soft-tissue neoplasms that do not metastasize, but exhibit aggressive growth and local invasion [5]. They are derived typically from the abdominal wall, the bowel and its mesentery, or in extra-abdominal sites such as the chest wall, shoulder girdle, inguinal region, and neck [6]. The etiology of DTs is unclear. Possible risk factors for their development include being female and/or having a previous history of surgery, trauma, or pregnancy [7]. In our case, the patient had an extra-abdominal DT and she had no identifiable risk factors associated with the condition. DTs can be categorized into superficial and deep subtypes. Deep-seated DTs are alsoreferred to as musculoaponeurotic subtypes. As stated above, DTs adjacent to osseous structures occasionally create a pressure effect and erode the cortex, as well as causing a spiculated periosteal reaction [3]. Pressure erosion is more typical with larger masses that are located near bone; however, it has been claimed that the osseous invasion of DTs can be facilitated through muscle attachments to bones [3]. In the present case, the DT, which involvedthe supraspinatus muscle, invaded the clavicle, despite the small size of the tumor.

For DTs, which arise from soft tissue, MR imaging is the most appropriate imaging modality to assist with diagnosis and to evaluate the margins and extent of the disease [8]. The appearance of DTs can vary from hypo-intense to hyper-intense, in comparison to muscle, and overall, the signal is heterogeneous [8]. There is no standard enhancement pattern after injection of gadolinium. DTs may have a heterogeneous signal and an inhomogeneous enhancement because of the variable distribution of spindle cells, collagen, and myxoid matrix. In radiography, DTs appear as ill-defined masses or soft-tissue swellings. On ultrasound, DTs appear as hypoechoic soft-tissue masses with variable vascularity [8]. The treatment of neoplasms such as DTs is guided by their clinical and evolutive characteristics. Radical therapy consists of wide resections of the tumor and adjoining tissuesresections [9]. As stated earlier, in pediatric cases, DTs are usually more infiltrative, havinga tendency for osseous involvement, and also have an earlier recurrence than in adult patients [10]. Cortical erosion was evident in our patient's shoulder radiograph, with cortical scalloping and irregularity of the osteolytic lesion located at distal 1/3 of the left clavicle (Figure A).

## Conclusion

This case illustrated the unusual features that can be associated with a soft-tissue desmoid tumor; namely, cortical disruption and medullary extension of the clavicle in an adult patient with a relatively small tumor.
